# Functional instant tea from black tea waste fiber: effects of extraction conditions on bioactive composition and *in vitro* anticancer activity

**DOI:** 10.3389/fnut.2026.1861937

**Published:** 2026-06-30

**Authors:** Neriman Ezgi Cifte, Gokhan Gorgisen, Emre Taskın, Beyza Bızıkcı, Buse Sonmez, Serap Namli, Mecit Halil Oztop

**Affiliations:** 1 Department of Food Engineering, Middle East Technical University, Ankara, Türkiye; 2 Department of Medical Biology, Gülhane Faculty of Medicine, University of Health Sciences, Ankara, Türkiye; 3 Dogadan Food Products Industry and Marketing Inc., Ankara, Türkiye; 4 METU Centre of Excellence in Biomaterials and Tissue Engineering (BIOMATEN), Ankara, Türkiye

**Keywords:** anticancer activity, bioactive compounds, black tea waste fiber, catechins, cold extraction, hot extraction, instant tea, MTT assay

## Abstract

**Introduction:**

Black tea processing generates large amounts of waste fiber that still retains considerable levels of bioactive compounds. In this study, black tea waste fiber was valorized for the production of instant tea powders, and the influence of extraction conditions on both bioactive composition and anticancer activity was investigated.

**Methods:**

Instant tea powders were produced using hot extraction (100 °C, 6 min) and cold extraction (25 °C, 2 h). Catechins, gallic acid, caffeine, theanine, theaflavin and thearubigins were quantified. Anticancer activity was evaluated by the MTT assay using prostate cancer cell lines (PC3, 22Rv1 and LNCaP), glioblastoma cell lines (U87MG and A172) and the breast cancer cell line MCF7.

**Results:**

Hot extraction resulted in significantly higher levels of catechins, condensed phenolics and theanine (*p* < 0.05). In contrast, the extended extraction time applied in the cold method partially compensated for mass transfer limitations in the extraction of gallic acid and caffeine. The anticancer potential of the instant teas was evaluated in vitro using prostate cancer cell lines (PC3, 22Rv1, and LNCaP), glioblastoma cells (U87MG and A172), and breast cancer cells (MCF7). The hot-extracted instant tea infusion inhibited the proliferation of prostate cancer cells in a concentration-dependent manner (*p* < 0.05). Hot-extracted instant tea showed a particularly strong cytotoxic effect on U87MG glioblastoma cells, reducing cell viability to 2.7% at 1.25 mg/mL, whereas A172 cells exhibited greater resistance. In contrast, cold-extracted samples demonstrated stronger inhibitory effects on MCF7 breast cancer cells (*p* < 0.05).

**Discussion:**

These findings demonstrate that black tea waste fiber can serve as a valuable raw material for functional instant tea production and highlight the importance of extraction conditions in shaping both the bioactive profile and biological activity of instant tea products derived from tea processing residues.

## 1 Introduction

**Figure 1:**
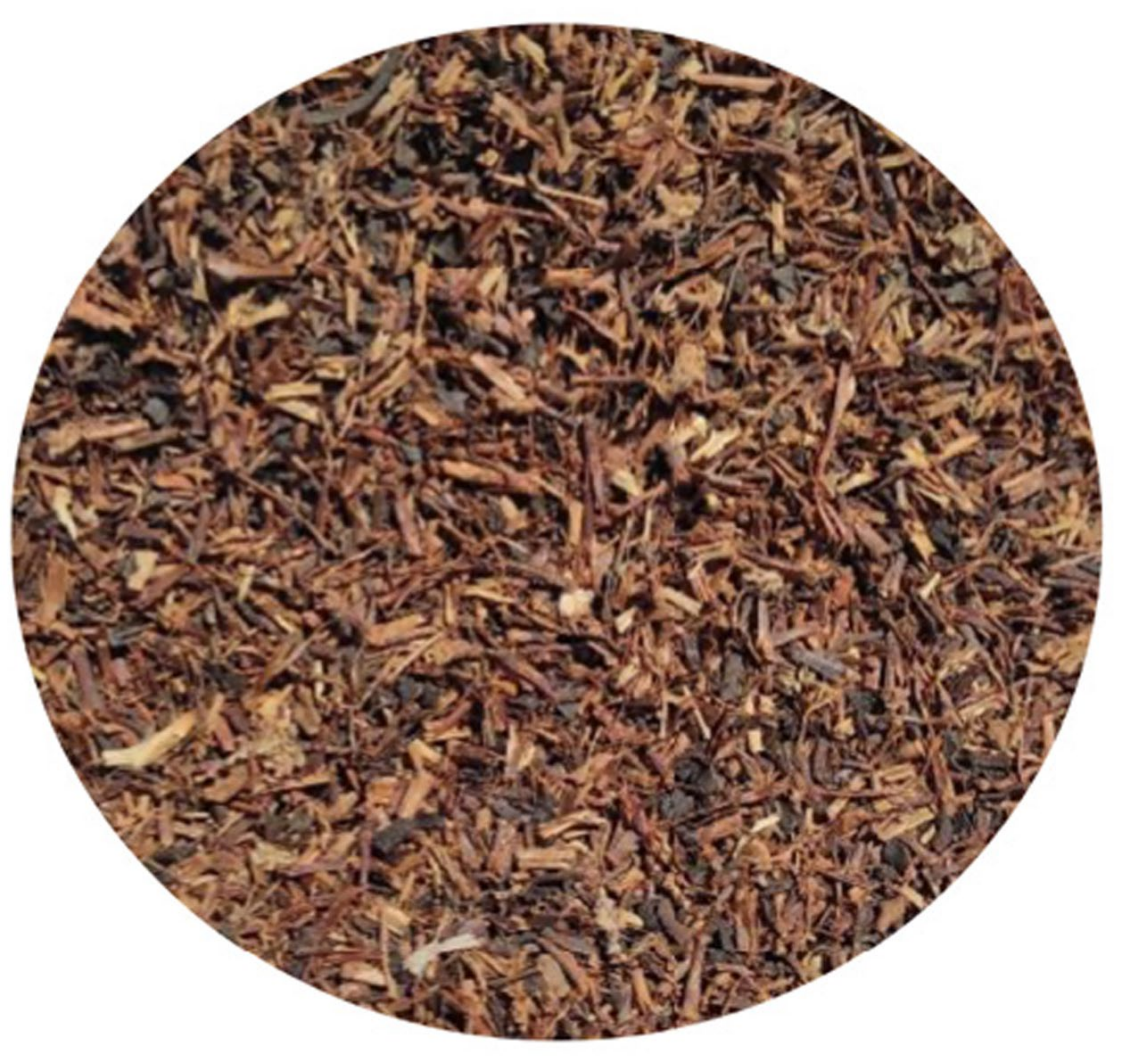
Representative image of black tea waste fiber used as the raw material for instant tea powder production.

In response to the growing demand for black tea, its production has increased considerably and is projected to expand at an annual rate of 2.1% by 2030 (1, 2). During the processing stages of black tea production, including withering, rolling, fermentation and drying, a significant amount of waste tea is inevitably generated (3). It has been reported that approximately 20–200 million kilograms of black tea waste are collected annually during harvesting and processing stages (4). This waste material mainly consists of lignocellulosic biomass composed of discarded tea leaves, stems and stalks (Figure 1) (4). Although these materials are generally considered processing residues, they still retain substantial amounts of biologically active compounds similar to those found in conventional tea (3). Such compositional retention is relevant because black tea waste fiber is separated from the end product after the main processing steps and contains not only fibrous fractions, such as discarded leaves, stems and stalks, but also a lignocellulosic matrix composed of cellulose, hemicellulose, lignin, polyphenols and tannins (4). In addition, tea waste has been reported to act as a reservoir of tea-derived bioactive constituents, including catechins, flavonoids, caffeine, theobromine, vitamins, minerals, terpenoids and amino acids (3). Therefore, although its composition differs from that of conventional tea products due to the higher proportion of coarse fibrous material, black tea waste fiber still represents a chemically valuable raw material for functional ingredient recovery.

Instant tea, a new-generation fully soluble black tea product, has been developed to meet changing consumer preferences and the growing demand for convenient beverage products (5). Extraction represents one of the most critical steps in the manufacturing of soluble tea, as the composition of the tea extract directly influences both the nutritional value and sensory characteristics of the final product (6, 7). While conventional hot extraction is widely used in instant tea production, cold extraction has recently emerged as a promising alternative due to its potential to enhance the extraction efficiency of certain bioactive compounds (8–11).

The bioactive compounds present in black tea, which also contribute to the value of tea waste, mainly consist of phenolic compounds such as catechins, including epicatechin (EC), epicatechin gallate (ECG), epigallocatechin (EGC) and epigallocatechin gallate (EGCG), as well as theaflavins, thearubigins and gallic acid, together with caffeine and theanine (3). The characteristic astringent flavor of tea products is largely attributed to variations in catechin composition (12). During black tea processing, catechins undergo enzymatic oxidation and polymerization reactions, resulting in the formation of complex polyphenolic compounds such as theaflavins and thearubigins (13). These compounds are responsible for the distinctive sensory attributes of black tea, including its reddish-brown color, brightness and briskness (14). In addition, gallic acid, caffeine and theanine, which are the most abundant phenolic acid, alkaloid and free amino acid in tea, respectively, also contribute significantly to the sensory profile of black tea (15, 16). Beyond their sensory contributions, these compounds have also been associated with several potential health benefits, including protective effects against neurological and cardiovascular diseases (16–18, 96). Recent studies further indicate that the chemical profile and bioactivity of black tea are influenced by processing and infusion-related conditions, including fermentation-induced changes in polyphenols and antioxidant bioactivity and infusion-dependent dissolution of functional components (19, 20). Among the reported health-related properties of tea-derived compounds, their anticancer potential has attracted particular attention due to the global burden and severity of cancer (21).

According to the World Health Organization, the global burden of cancer is expected to reach approximately 27 million cases and 17.5 million deaths annually by 2050 (22). Prostate cancer represents one of the leading causes of cancer-related mortality worldwide and is the second most frequently diagnosed cancer among men after lung cancer (23). In addition, glioblastoma multiforme (GBM) is recognized as the most common and aggressive tumor of the central nervous system, with an average survival time of fewer than 15 months (24). Breast cancer also represents a major global health challenge as the most frequently diagnosed cancer worldwide (25).

The selected cancer cell lines were chosen to cover cell models with different biological characteristics and treatment responses. PC3, 22Rv1, and LNCaP were included to represent prostate cancer cell lines with different androgen responsiveness and genetic backgrounds (26). U87MG and A172 were selected as glioblastoma models because they differ in genetic alterations and cytotoxic response patterns, including PTEN mutation in both cell lines and additional TP53 mutation-associated resistance in A172 cells (27). MCF7 was included as a commonly used breast cancer cell model to evaluate the response of breast cancer cells to the instant tea samples.

Various conventional treatment strategies, including immunotherapy, hormone therapy, radiation therapy, chemotherapy and surgical interventions, have been widely employed to reduce cancer-related mortality. However, these therapeutic approaches are often associated with severe adverse side effects in patients (28). Consequently, increasing attention has been directed toward the identification of novel anticancer agents derived from natural sources that may exhibit cytotoxic activity with potentially fewer side effects (26). In this context, several studies have demonstrated that tea extracts and tea-derived bioactive compounds possess cytotoxic and antiproliferative activities against different cancer cell lines (29–32).

In our previous study, the feasibility of producing instant tea from black tea waste fibers was demonstrated, and the resulting products were characterized in terms of their physicochemical properties and bioactive composition (33). However, most existing tea studies have focused on conventional tea leaves, commercial tea infusions, or isolated tea-derived compounds, while studies on tea processing residues have mainly addressed waste valorization, extraction efficiency, antioxidant properties, or compositional characterization. Therefore, the biological functionality of instant tea powders produced directly from black tea waste fibers remains insufficiently understood. In particular, the anticancer activity of black tea waste fiber-derived instant teas and the extent to which hot and cold extraction conditions modify both their bioactive composition and cancer cell responses have not been systematically investigated. The novelty of the present study is that it integrates three aspects that have generally been examined separately: valorization of black tea waste fiber into instant tea powder, extraction-dependent profiling of major tea bioactives, and evaluation of *in vitro* anticancer activity across prostate cancer, glioblastoma, and breast cancer cell models. This integrated approach provides new insight into whether an underutilized tea-processing residue can be converted into a functional instant tea product with biologically relevant activity.

Therefore, the present study aimed to develop instant tea powders from black tea waste fiber and to investigate the effects of hot-extracted instant tea infusion (HI) and cold-extracted instant tea infusion (CI) on bioactive composition and in vitro anticancer activity against prostate cancer (PC3, 22Rv1, and LNCaP), glioblastoma (U87MG and A172) and breast cancer (MCF7) cell lines.

## 2 Materials and methods

### 2.1 Materials

Black tea waste fiber, consisting of residual fibrous material generated during industrial black tea processing, was supplied by Doğadan Tea Company (Ankara, Türkiye) in 2021. The material originated from black tea produced in Türkiye using the Orthodox processing method and was obtained from tea harvested between April and October. The initial moisture content of the supplied black tea waste fiber was approximately 4%. Specific cultivar information was not available because the material was supplied as a mixed industrial processing residue. The waste fiber was used in its supplied dry form without any additional washing or sieving step before milling. Owing to its irregular structure and heterogeneous particle size, the material was directly subjected to high-shear grinding and subsequent planetary ball milling prior to extraction.

All chemicals and reagents were purchased from Sigma-Aldrich Chemical Company (St. Louis, MO, USA), except for RPMI-1640 medium, DMEM medium, HEPES, penicillin–streptomycin and sodium pyruvate acquired from Thermo Fisher Scientific (Grand Island, NY, USA).

### 2.2 Production of instant black tea powders

Black tea waste fibers were first reduced in size by high-shear grinding (Vorwerk Thermomix GmbH, Wuppertal, Germany) at 10,700 rpm for 3 min, followed by planetary ball milling (Liya Laboratory Testing Equipment Co., Ltd., Ankara, Türkiye) at 600 rpm for 15 min. The 15 min ball-milling condition was selected based on previously optimized production parameters; under these conditions, the pre-ground and ball-milled fiber had a particle size range of 4.12–76.02 μm and a mean particle size of 19.39 μm, while avoiding the higher temperature increase associated with longer dry milling. For extraction, the pulverized waste fibers were infused with distilled water at a water-to-tea ratio of 30:1 mL/g. Hot extraction was conducted at 100 °C for 6 min with 15 s mixing every 3 min, whereas cold extraction was conducted at 25 °C for 2 h with 15 s mixing every 30 min. The selected water-to-tea ratio, particle size, extraction temperature and extraction time were based on previously optimized conditions for black tea waste fiber-derived instant tea production and were kept constant for all samples to ensure reproducibility (33). The extracts were then separated using 10 μm polypropylene filters and frozen at −18 °C for 24 h before freeze-drying (Beijing Songyuan Huaxing Technology Development Co., Ltd., Beijing, China) for 48 h. The freeze-drying process was continued until the final moisture content of the instant tea powders reached 4.0–4.5% on a wet basis. Moisture content was determined gravimetrically by drying the samples in an oven at 105 °C until constant weight.

### 2.3 Quantification of catechins, gallic acid, caffeine, and theanine

EC, ECG, EGC, EGCG, gallic acid, caffeine and theanine were quantified by HPLC according to Serpen et al. (16), with modifications. Analytical standards of epicatechin, epicatechin gallate, epigallocatechin, epigallocatechin gallate, gallic acid, caffeine and L-theanine were used for compound identification and quantification. The compounds were identified by comparing the retention times and absorption spectra of sample peaks with those of the corresponding standards, and quantification was performed using external calibration curves prepared for each standard compound. A sample of 500 mg of instant tea was extracted with 5 mL of distilled water at 80 °C. The mixture was stirred for 5 min and then centrifuged at 9,055 g for 5 min using a centrifuge (Wisd Laboratory Instruments Co., Ltd., Dusseldorf, Germany). The supernatant was collected, and the extraction was repeated once for the residue under the same conditions. The combined extract solutions were filtered through a 0.45 μm nylon filter and transferred to HPLC vials. The extracted catechins, gallic acid, caffeine and theanine were quantified using an HPLC system (Shimadzu Scientific Instruments Co., Ltd., Kyoto, Japan) equipped with a DG-20A5 degasser, LC-20 AD pump, SIL-20AHT autosampler, CTO-20A column oven and SPD-M20A photodiode array detector. The chromatographic conditions were as follows: Hypersil GOLD Phenyl HPLC column (250 × 4.6 mm, 5 μm); UV detection was 275 nm; mobile phase A, acetonitrile, mobile phase B, 0.1% (v/v) aqueous acetic acid; flow rate, 1 mL/min; temperature, 25 °C; injection volume, 10 μL; gradient elution: 10–20% A, 0–15 min; 20–40% A, 15–25 min; 40–10% A, 25–30 min. For theanine quantification, the chromatographic conditions were arranged as follows: UV detection was 200 nm; mobile phase A, acetonitrile, mobile phase B, water; flow rate, 1 mL/min; temperature, 25 °C; injection volume, 10 μL; gradient elution: 2% A, 0–5 min; 2–50% A, 5–12 min; 50% A, 12–30 min; 50–2% A, 30–40 min; 2% A, 40–45 min (34).

### 2.4 Quantification of theaflavin and thearubigins

The content of theaflavin and thearubigins in instant black teas was determined using a modified Flavognost method (35, 36). A tea infusion with a concentration of 24% (w/v) was prepared using freshly boiled distilled water and allowed to cool to room temperature. Then, 10 mL of the tea infusion was mixed with 10 mL of isobutyl methyl ketone (IBMK) using a shaker at 300 rpm for 10 min (Daihan Scientific Co., Ltd., South Korea). After phase separation, 2 mL of the upper layer was mixed with 4 mL of ethanol and 2 mL of Flavognost reagent (prepared by dissolving 2 g of diphenyl boric acid-2-aminoethyl ester in 100 mL of ethanol). The absorbance was measured at 625 nm against an IBMK:ethanol (1:1, v/v) blank after allowing it to stand for 15 min. The theaflavin content was calculated using Equation (1), adapted from the modified Flavognost method (35, 36):


$$\begin{array}{l} \text{Theaflavin}\;(\mathrm{mg}/100\;g\;\mathrm{dry}\;\text{matter})\\ =\frac{\text{Absorbance}\times 47.9\times 10\times 564.49}{\mathrm{Dry}\;\text{matter}\;(\%)} \end{array}$$ (1)

where the absorbance value corresponds to the measurement at 625 nm, 47.9 is the dilution and correction factor used in the Flavognost method, 10 is the conversion factor for expressing the result per 100 g of sample, 564.49 g/mol is the molecular weight of theaflavin, and dry matter (%) represents the dry matter content of the instant tea powder.

The same tea infusion was used for the thearubigins quantification, where 10 mL of the solution was first mixed with 50 mL of IBMK, shaken at 300 rpm for 10 min, and left for phase separation. Then, 4 mL of the upper layer was diluted to 25 mL with methanol (solution A). Moreover, 25 mL of the upper layer was mixed with 25 mL of 2.5% aqueous sodium bicarbonate solution, and 4 mL of this mixture was diluted to 25 mL with methanol (solution B). Furthermore, 2 mL of the upper layer was combined with 2 mL of saturated oxalic acid solution and 6 mL of distilled water, and the mixture was diluted to 25 mL with methanol (solution C). The absorbance values of solutions A, B, and C were read at 380 nm against a distilled water blank. The thearubigins content was calculated using Equation (2), adapted from the modified Flavognost method (35, 36):


$$\begin{array}{l} \text{Thearubigins}\;(\mathrm{mg}/100\;g\;\mathrm{dry}\;\text{matter})\\ =\frac{100000\times 0.02\times 6.25\times (2\times {\mathrm{Abs}}_{C}+{\mathrm{Abs}}_{A}-{\mathrm{Ab}}_{B})}{0.733\times 0.024\times \mathrm{Dry}\;\text{matter}\;(\%)} \end{array}$$ (2)

where AbsA, AbsB, and AbsC represent the absorbance values of solutions A, B and C at 380 nm, respectively; 100,000, 0.02 and 6.25 are dilution and unit-conversion factors used to express the result as mg/100 g dry matter; 0.733 and 0.024 are empirical coefficients used in the Flavognost calculation; and dry matter (%) represents the dry matter content of the instant tea powder.

### 2.5 Cell culture

The anticancer activities of instant teas were investigated using prostate cancer cell lines PC3 (ATCC® CRL-1435™), 22Rv1 (ATCC® CRL-2505™) and LNCaP (ATCC® CRL-1740™), glioblastoma multiforme cell lines U87MG (ATCC® HTB-14™) and A172 (ATCC® CRL-1620™), and the breast cancer cell line MCF7 (ATCC® HTB-22™). PC3, 22Rv1 and LNCaP cells were maintained in RPMI-1640 medium supplemented with 10% fetal bovine serum, 1% penicillin–streptomycin, nonessential amino acids, sodium pyruvate and HEPES (4-(2-hydroxyethyl)-1-piperazine ethanesulfonic acid). U87MG, A172, and MCF7 cells were cultured in DMEM medium supplemented with 10% fetal bovine serum, 1% penicillin–streptomycin, nonessential amino acids and sodium pyruvate. All cells were grown at 37 °C in a humidified incubator with 5% CO₂ and were subcultured at approximately 75–80% confluence before use in the viability assay.

### 2.6 MTT assay

The viability of the cell lines was determined using the tetrazolium microculture assay (MTT, 3-(4,5-dimethylthiazol-2-yl)-2,5-diphenyltetrazolium bromide), a colorimetric method used to evaluate cell viability and metabolic activity based on the reduction of the tetrazolium salt MTT to insoluble formazan crystals by metabolically active cells. Before seeding, cells were counted using a hemocytometer to adjust the required cell density. For the analysis, cells were seeded in 96-well plates at a density of 4,000 cells per well in 100 μL of growth medium and incubated overnight. The MTT assay was performed with four replicate wells for each treatment condition. The cells were then treated with varying concentrations of instant tea solutions (0.001, 0.01, 0.1, 1, 1.25, 2.5, 5, 8, and 10 mg/mL), which were freshly prepared before treatment by dissolving the samples in isotonic solution at 95 °C for 15 min and filtering through a 0.22 μm filter. This dissolution step was applied to ensure complete solubilization of the freeze-dried instant tea powders and to obtain particle-free solutions suitable for sterile filtration before cell treatment. Although heat-sensitive compounds may be affected during sample preparation, the same dissolution procedure was applied consistently to all instant tea samples prior to the MTT assay. Therefore, the observed cytotoxic responses reflect the activity of the final treated solutions prepared under standardized conditions. After 48 h incubation, 20 μL of MTT solution (5 mg/mL in PBS) was added to each well and cultured for 4 h at 37 °C. The supernatant was aspirated, and MTT formazan crystals, formed by metabolically viable cells, were dissolved in 100 μL of dimethyl sulfoxide solution. The absorbance values were monitored at 560–620 nm with a microplate reader. Cell viability was calculated relative to the untreated control group and expressed as a percentage using the following formula: cell viability (%) = (Absorbance of treated cells / Absorbance of control cells) × 100.

### 2.7 Statistical analysis

Experimental results were presented as mean ± standard deviation. Statistical analysis was performed by analysis of variance (ANOVA) with Tukey’s Comparison Test in MINITAB (Version 20, Minitab Inc., UK) at the confidence interval of 95%.

## 3 Results and discussion

### 3.1 Analysis of phenolics, caffeine and theanine

#### 3.1.1 Overview of bioactive composition

The phenolic profile, including four main flavanols (EC, ECG, EGC, and EGCG), two condensed phenolics (theaflavin and thearubigins), one phenolic acid (gallic acid), as well as caffeine and theanine contents of instant tea samples produced by hot and cold extraction of black tea waste fibers, is presented in Table 1.

**Table 1:** Phenolics, caffeine and theanine contents of instant teas.

Sample	EC (mg/100 g)	ECG (mg/100 g)	EGC (mg/100 g)	EGCG (mg/100 g)	Theaflavin (mg/100 g)	Thearubigins (mg/100 g)	Gallic Acid (mg/100 g)	Caffeine (mg/100 g)	Theanine (mg/100 g)
HI	**3,348.3 ± 49.2** ^**a**^	**359.9 ± 11.9** ^**a**^	**1,502.8 ± 4.6** ^**a**^	**249.5 ± 14.7** ^**a**^	**244.7 ± 1.9** ^**a**^	**2,593.2 ± 9.6** ^**a**^	1,122.6 ± 9.6^a^	3,410.5 ± 26.5^a^	**1,368.1 ± 0.5** ^**a**^
CI	2,931.3 ± 105.3^b^	283.0 ± 9.5^b^	1,012.0 ± 19.4^b^	162.1 ± 2.7^b^	120.3 ± 1.9^b^	1,630.7 ± 5.0^b^	1,101.1 ± 17.7^a^	3,434.7 ± 26.5^a^	1,201.7 ± 21.7^b^

Different letters in the same column represent a significant difference (*p* < 0.05). Bold values indicate the most prominent findings discussed in the text.

#### 3.1.2 Catechin and condensed phenolic profile

EC was identified as the most abundant catechin in the instant tea samples, followed by EGC, ECG, and EGCG. Although the instant teas were produced from black tea waste fibers, their ECG (428.00 and 315.00 mg/100 g) and EGCG (176.00 and 173.00 mg/100 g) contents were similar to those reported previously for instant black teas produced from conventional tea samples (37). The theaflavin value of instant tea produced by the hot extraction method (HI) was higher than the literature values of 56.00 and 124.00 mg/100 g reported by Alasalvar et al. (37) and 165.00 and 175.00 mg/100 g reported by Someswararao et al. (38), unlike the cold extraction method (CI). Thearubigins levels of the current study were similar to the findings of Someswararao and Srivastav (38), 1,973 and 4,526 mg/100 g, while lower than those of Alasalvar et al. (37), 10,161 and 12,701 mg/100 g. Alasalvar et al. (37) also stated lower gallic acid, 697.00 and 751.00 mg/100 g, and theanine, 512.80 and 572.20 mg/100 g, values and higher caffeine, 3,964 and 4,398 mg/100 g, for their instant tea samples. The relatively high levels of several bioactive compounds observed in the instant tea powders suggest that black tea waste fibers still retain substantial amounts of functional phytochemicals after industrial processing. This finding further supports the potential of tea processing residues as valuable raw materials for the development of functional tea products.

#### 3.1.3 Comparison with conventional black tea and instant tea literature

Comparable levels were also observed in conventional black teas for ECG, 89.5–115.0 mg/100 g (16) and 214.00–767.00 mg/100 g dry matter (39), EGC, 1,038.0–1,214.0 mg/100 g (16) and 502.00–2,203.00 mg/100 g dry matter (39), EGCG, 102.0–155.0 mg/100 g (16) and 304.0–1,540.0 mg/100 g dry matter (39), and theaflavins, 121.00–399 mg/100 g (16) and 0–540 mg/100 g (40). In addition, thearubigins contents of conventional black teas were found to be 5,920.00–6,830 mg/100 g (16) and 8,580–11,970 mg/100 g (36). Although no obvious pattern could be seen for the others, EC [362.86 mg/100 g (41); 263.00–340.00 mg/100 g dry matter (36)], gallic acid [250.00–450.00 mg/100 g (42); 96.80–116.00 mg/100 g (16)], caffeine [2,206.99 mg/100 g (41); 1,348–2,344 mg/100 g (43)] and theanine [513.00 mg/100 g (43); 480–1,190 mg/100 g dry matter (44)] contents of the powders needed special attention because of their higher levels. The variation observed in the profile of bioactive compounds may be attributed to several factors, including tea variety, cultivation conditions, processing parameters and extraction conditions, all of which are known to influence the release and stability of tea polyphenols (10, 16, 36, 45).

#### 3.1.4 Effect of extraction conditions

It was determined that HI had higher catechins, condensed phenolics and theanine contents than instant tea produced by CI (*p* < 0.05). A similar pattern was also observed in the study of Yang et al. (11), where catechin extraction in black tea at 100 °C for 4 min was achieved more efficiently than at 25 °C for 16 h. Increasing extractability of tea bioactive compounds with the rising temperature could be explained by the enhancing effect of temperature on the permeability of plant cell walls, mass transfer, diffusion coefficients and solubility (11, 46–48). In addition, elevated temperatures may facilitate the disruption of plant cell structures, allowing intracellular polyphenols to diffuse more readily into the extraction medium.

#### 3.1.5 Interpretation of gallic acid and caffeine extraction

On the other hand, the high temperature could not improve the extraction of gallic acid and caffeine over the cold extraction method (*p* > 0.05). Thus, it was proposed that the extended extraction period of the cold extraction could compensate for the mass transfer rate limitation in the extraction of gallic acid and caffeine (49). Therefore, although cold extraction occurs at lower temperatures, prolonged extraction time may partially overcome mass transfer limitations and allow the gradual diffusion of certain compounds such as caffeine and gallic acid into the solvent. Damiani et al. (50) also suggested this trend in their research for caffeine extraction in white teas, where extraction at 25 °C for 2 h led to greater extraction than at 70 °C for 7 min. These results indicate that black tea waste fibers retain considerable levels of bioactive compounds even after industrial processing.

### 3.2 Analysis of anticancer activities

#### 3.2.1 Prostate cancer cells (PC3, 22Rv1, and LNCaP)

HI was selected for the anticancer activity confirmation in prostate cancer cell lines because it contained higher levels of catechins, condensed phenolics and theanine than CI. Therefore, this part of the experiment was designed to confirm the cytotoxic potential of the bioactive-rich instant tea infusion, whereas the comparative effect of extraction conditions on anticancer activity was subsequently evaluated using U87MG, A172, and MCF7 cells.

**Figure 2:**
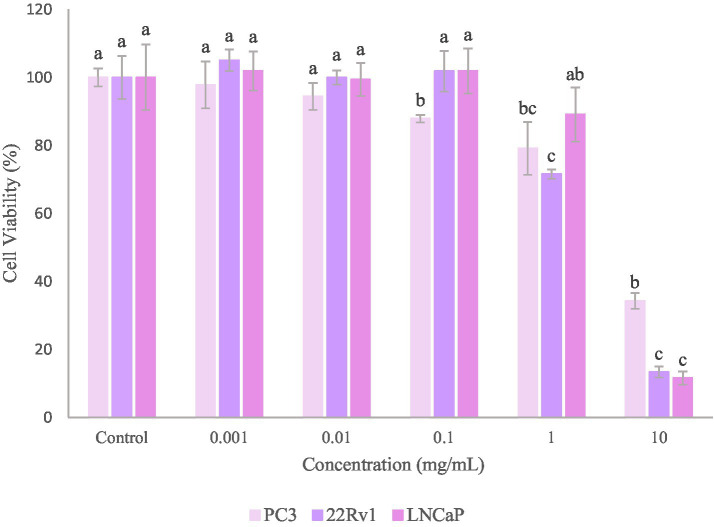
Cell viability of prostate cancer cell lines (PC3, 22Rv1, and LNCaP) after 48 h treatment with HI. Data are expressed as mean ± standard deviation. Different letters indicate statistically significant differences compared with the untreated control at the same concentration (*p* < 0.05).

The cell viabilities of prostate cancer cells against HI are presented in Figure 2. The results demonstrated that instant tea extracts suppressed the viability of all tested prostate cancer cells in a concentration-dependent manner (*p* < 0.05). Moreover, in the range of 0.1–10 mg/mL, the prostate cancer cells showed varying susceptibilities to the anticarcinogenic effects of instant teas (*p* < 0.05). The differences in cellular responses may be associated with the biological characteristics of the prostate cancer cell lines, including their androgen sensitivity and genetic background (26).

PC3, the androgen-independent prostate cancer cell, was determined as the most sensitive cell line because its viability was reduced at the concentration of 0.1 mg/mL, 87.8% (*p* < 0.05). In addition, 22Rv1, the androgen-independent but sensitive prostate cancer cell, started to decrease at the concentration of 1 mg/mL, 71.56% (*p* < 0.05). On the other hand, in the present study, LNCaP, the androgen-dependent prostate cancer cell, was considered the most resistant cell line because the cell viability inhibition was observed at 10.0 mg/mL, 11.54% (*p* < 0.05).

The study by Sun et al. (51) verified the anticancer potential of EGCG, known as the most effective component in tea against cancer cell survival, for PC3 cells in a concentration-dependent manner by MTT analysis (52). Moreover, Yang et al. (53) found that EGCG induced apoptosis and necrosis of PC3 cells by mitochondrial dysfunction, i.e., the mitochondria-mediated pathway. Apoptosis and necrosis are the main mechanisms of cell death, with apoptosis being a controlled autonomous cellular process and necrosis resulting from uncontrolled environmental damage (54). In the mitochondria-mediated pathway, proteins like cytochrome c are released into the cytosol due to increased permeability of the mitochondrial membrane, the mitochondrial membrane loses its potential, and ATP synthesis is inhibited. Sun et al. (55) also determined this pathway in PC3 cells treated with black tea extracts and theaflavins. Furthermore, these studies highlighted the activation of caspase 3 (55) and caspase 9 (53), enzymes responsible for apoptosis, due to the presence of EGCG, black tea extracts and theaflavins. Liu et al. (56) demonstrated a concentration-dependent inhibition of PC3 cell viability due to the presence of gallic acid. The anticancer mechanism of gallic acid involves inducing DNA damage and DNA repair by altering the expression of DNA repair genes such as ataxia telangiectasia mutated, ataxia-telangiectasia and Rad3-related, DNA-dependent serine/threonine protein kinase and p53 mRNA (56).

Alserihi et al. (26) also investigated the reactive oxygen species (ROS)-mediated pathway, where ROS causes cell death by generating oxidative stress in cells, in the cytotoxicity of EGCG in PC3 and 22Rv1 cell lines (57). They observed an increase in ROS formation in PC3 cells by EGCG, unlike in 22Rv1 cells. Moreover, the study showed a dramatic reduction in the sizes of cancer cell spheroids. This observation was essential since larger tumor sizes are associated with higher resistance (58). The decrease in spheroid size was significantly higher in PC3 cells compared to 22Rv1 cells, like the present study where 22Rv1 cells showed higher resistance to instant teas than PC3 cells. Unlike earlier studies (53, 55), the recent study (26) did not observe a significant loss of mitochondrial membrane potential in prostate cancer cells due to EGCG. Therefore, the researchers hypothesized that EGCG might also work through a mitochondrial-independent mechanism for anticancer activity. Rahman Siddique et al. (59) also reported a decrease in cell viability of 22Rv1 cells because of the ability of EGCG to reduce androgen receptor protein expression. Additionally, Kaur et al. (60) revealed the cytotoxicity of gallic acid against 22Rv1 cells by inducing apoptosis in 22Rv1 cells.

Eom et al. (61) found that EGCG had a stronger concentration-dependent inhibitory effect on LNCaP cells compared to PC3 cells, which contrasts with the present research on instant teas. Bakhshandeh et al. (62) also confirmed the cytotoxicity of EGCG by reducing the proliferation of LNCaP cells. Nam et al. (63) found that the ester bond in EGCG was responsible for its cytotoxicity on LNCaP cells by arresting the proteasome in the cell, leading to the accumulation of proteasome substrates, p27Kip1 and IκB-*α*, inhibiting the key, the transcription factor NF-κB, for cell proliferation and survival (64). It was also stated that EGCG stimulated AMP-activated protein kinase (AMPK), which is involved in apoptosis (62, 65). Lin et al. (66) also revealed the chemoreceptive potentials of theaflavins (theaflavin, theaflavin-3-gallate, theaflavin-3′-gallate, and theaflavin-3,3′-digallate) along with EGCG on LNCaP cells by suppressing 5α-reductase, an enzyme responsible for the progression of the cells, and inhibiting the expression of androgen receptors in LNCaP cells. Furthermore, Russell et al. (67) demonstrated that gallic acid reduced the cell viability of LNCaP cells through a ROS-mediated pathway.

#### 3.2.2 Brain tumor cells (U87MG and A172)

After the confirmation, the impact of hot and cold extraction methods on the anticancer activity was investigated on glioblastoma multiforme (GBM) cells, U87MG and A172.

**Figure 3:**
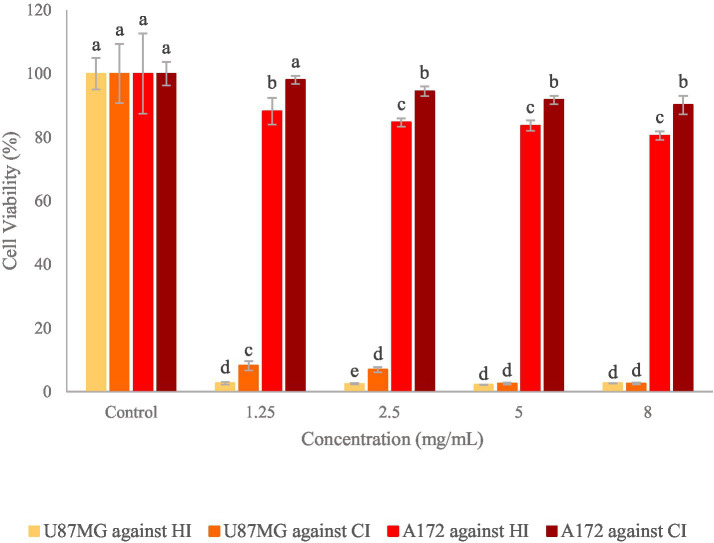
Cell viability of glioblastoma multiforme cell lines (U87MG and A172) after 48 h treatment with HI and CI. Data are expressed as mean ± standard deviation. Different letters indicate statistically significant differences compared with the untreated control at the same concentration (*p* < 0.05).

The cell viabilities of GBM cells treated with HI and CI are presented in Figure 3. Both instant teas had a concentration-dependent anticancer effect on U87MG and A172 cells (*p* < 0.05). In addition, HI decreased the cell viability of U87MG and A172 cells more than CI (*p* < 0.05), except for U87MG cells at 5 and 8 mg/mL (*p* > 0.05). This trend was consistent with the higher catechin and condensed phenolic contents of HI; however, the magnitude of the response differed markedly between U87MG and A172 cells, indicating a strong cell-line-dependent effect. The greater EGCG content in HI was especially noteworthy, as it was considered the most promising cytotoxic agent in tea (52, 68). Moreover, HI reduced the cell viability of U87MG cells to 2.7% and A172 cells to 88.1% at the concentration of 1.25 mg/mL (*p* < 0.05), with no significant difference at 2.5, 5 and 8 mg/mL (*p* > 0.05). On the other hand, CI significantly decreased the cell viability of U87MG cells to 8.2% at 1.25 mg/mL and 2.6% at 5 mg/mL and A172 cells to 94.5% at 2.5 mg/mL and 90.1% at 8 mg/mL (*p* < 0.05).

Instant teas had a higher anticancer effect on U87MG cells compared to A172 cells (*p* < 0.05), indicating that A172 cells were more resistant to instant teas. This difference in response of the cells is due to their genomic variations, such as genetic alterations that make glioblastoma cells more resistant to treatments (27). Both cells have genetic mutations in the phosphatase and tensin homolog deleted on chromosome ten (PTEN) gene, a tumor suppressor gene (27). The main function of PTEN is to dephosphorylate phosphatidylinositol (3,4,5)-trisphosphate (PIP3), activating the phosphatidylinositol-3 kinase (PI3K) pathway, a key signal transduction mechanism that controls cell growth and survival (69, 70). The mutation in PTEN increases PIP3 levels, leading to uncontrolled cell growth and tumor formation by stimulating the PI3K pathway (71). Furthermore, A172 cells have a mutation in the TP53 gene, which encodes the p53 protein, known as the “guardian of the genome” and regulates cellular responses like division and differentiation (27, 72, 73). When cells face stress signals, p53 is activated to initiate DNA repair, cell cycle arrest and apoptosis (74). However, the mutated p53 in A172 cells, known as the guardian of cancer cells, promotes cancer cell survival by allowing unrestricted cell cycles and preventing apoptosis (72, 73). Therefore, this mutation gives A172 glioblastoma cells extra resistance to cytotoxic agents (27).

EGCG-induced apoptosis in U87MG cells in a concentration-dependent manner by the ROS-mediated pathway was noted in several studies (75, 76). Das et al. (75) also indicated that EGC and EGCG activated caspase 8, triggering the mitochondria-mediated apoptosis in U87MG cells. Moreover, Shervington et al. (77) found that EGCG inhibited cell proliferation and telomerase protein, linked to the malignancy level in U87MG cells, ultimately leading to apoptosis. These mechanisms are frequently associated with the activity of tea polyphenols, particularly catechins and theaflavins, which have been reported to influence mitochondrial function and apoptosis pathways in various cancer cell models. On the other hand, Yang et al. (78) confirmed that gallic acid decreased U87MG cell viability in a concentration-dependent manner. They also suggested that the cytotoxic effect of gallic acid could be based on p38 MAPK (mitogen-activated protein kinase) activation, involved in cell cycle regulation and apoptosis. Furthermore, the concentration-dependent anticancer activity of caffeine was shown in the study of Li et al. (79).

EGCG-induced apoptosis in the aggressive glioblastoma cells of A172 was demonstrated in the study of Siegelin et al. (80), where it was found that EGCG suppressed apoptosis preventing surviving proteins and phosphoprotein enriched in astrocytes (PEA15), known as tumor necrosis factor-related apoptosis-inducing ligand (TRAIL)-mediated pathway. TRAIL has a strong ability to activate apoptosis in cancer cells (81). Moreover, Sachinidis et al. (82) reported the anticancer activity of ECG and EGCG on A172 cells by inhibiting the activity of the platelet-derived growth factor receptor *β* (PDGF-Rβ) (83).

#### 3.2.3 Breast cancer cell (MCF7)

The anticancer behaviors of HI and CI were also shown on the breast cancer cell, MCF7.

**Figure 4:**
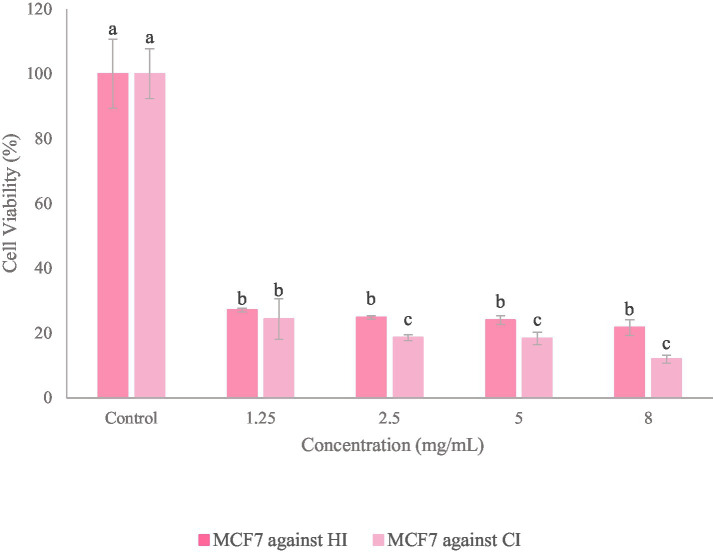
Cell viability of MCF7 breast cancer cells after 48 h treatment with HI and CI. Data are expressed as mean ± standard deviation. Different letters indicate statistically significant differences compared with the untreated control at the same concentration (*p* < 0.05).

The cell viability of the breast cancer cells treated with HI and CI is presented in Figure 4. Instant teas showed concentration-dependent cytotoxic effects on MCF7 cells (*p* < 0.05). CI decreased the cell viability of MCF7 cells more than HI (*p* < 0.05), except at 1.25 mg/mL (*p* > 0.05). This unexpected result suggested that other bioactive molecules in CI, besides catechins, theaflavin, thearubigins and theanine, found higher in HI, could be responsible for their anticancer activities by suppressing the effects of the greater phenolic composition of HI. It was also revealed that Camellia tenuifolia had the highest apoptosis induction ability on MCF7 cells among the six different Camellias species, following *Camellia sinensis*, despite its lowest catechin content in the study of Way et al. (84). Then, they concluded that the cytotoxicity of Camellia tenuifolia was caused by other bioactive components instead of catechins. In addition, the interactions between components during extractions could affect the cytotoxic ability of instant teas on MCF7 cells. It was also supported in the study of Friedman et al. (40) by finding that competitive and antagonistic interactions among the flavonoids could contribute to the anticancer activity of commercial black teas as catechin and theaflavin levels did not correlate with the cytotoxicity of the tea samples. Moreover, the morphological structures of HI and CI, caused by different extraction conditions, could affect their anticancer abilities, such as cellular uptake (85–87).

HI and CI started to decrease MCF7 cell viability at 2.5 mg/mL (*p* < 0.05). Moreover, HI reduced the cell viability of MCF7 cells to 27.1% at 1.25 mg/mL and 23.9% at 5 mg/mL (*p* < 0.05). On the other hand, CI decreased the cell viability of MCF7 cells to 24.3% at 1.25 mg/mL and 11.9% at 8 mg/mL (*p* < 0.05).

**Figure 5:**
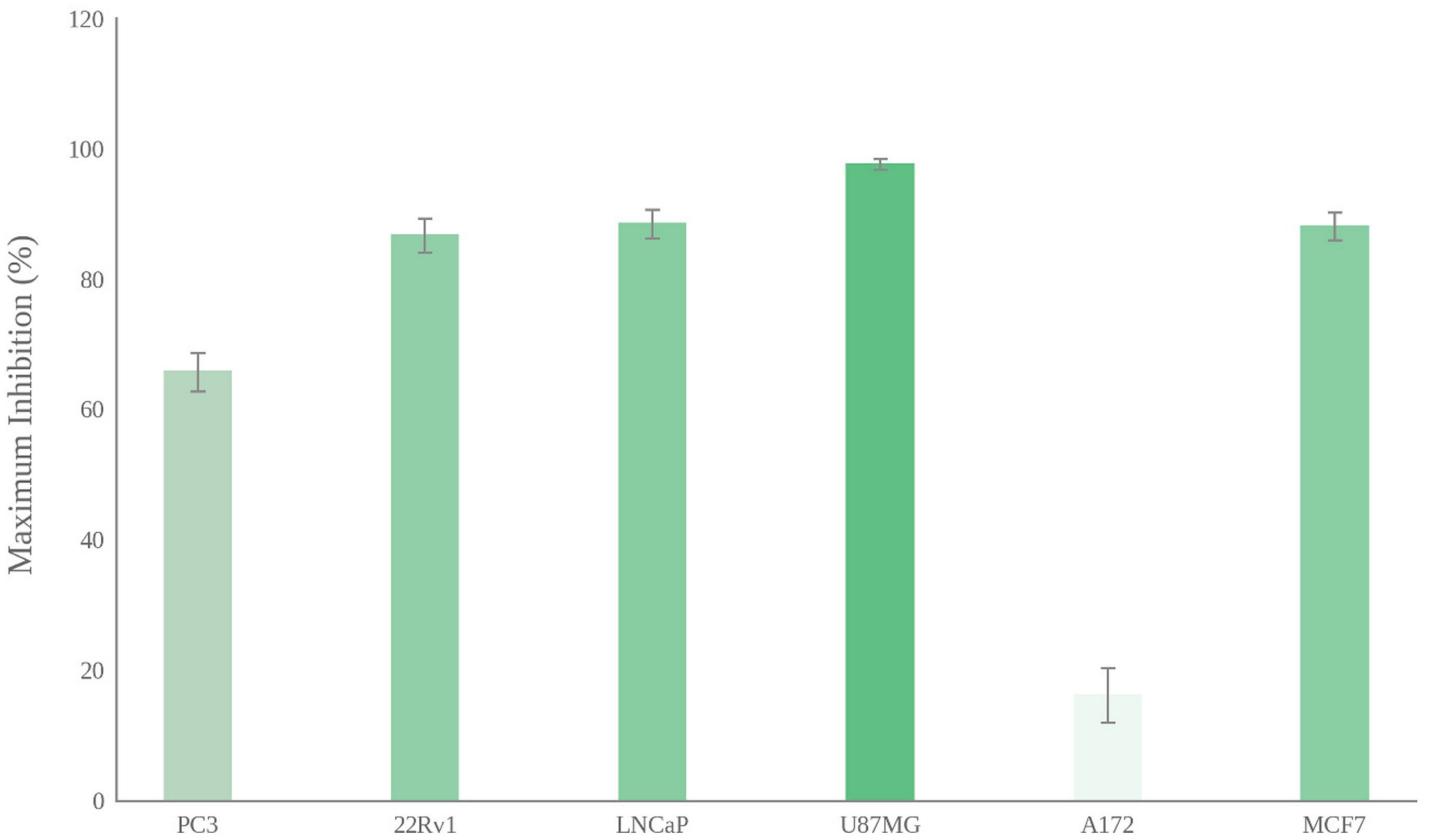
Comparative maximum inhibition across the tested cancer cell lines after treatment with instant tea samples. Values represent inhibition (%) calculated from the lowest cell viability responses.

To provide an overall comparison of cell-line sensitivity, the maximum inhibition values calculated from the lowest comparable cell viability responses are summarized in Figure 5. Overall, the comparative summary highlights the cell-line-dependent response to instant tea treatments, with U87MG, LNCaP, MCF7 and 22Rv1 showing greater inhibition than A172 under the tested conditions.

Way et al. (84) also found that fresh tea leaf extract decreased the cell viability of MCF7 cells in a concentration-dependent manner. They observed that fresh tea leaves induced apoptosis in MCF7 cells through the activation of caspase 3, which cleaved two apoptosis proteins, poly-ADP ribose polymerase (PARP) and BH3 interacting domain death agonist (BID). Koňariková et al. (88) also confirmed the cytotoxicity of black tea on MCF7 cells by causing DNA strand breaks and oxidative DNA damage. The anticancer activities of commercial black teas against MCF7 cells were also supported by the research of Friedman et al. (40), where the concentration-dependent cytotoxicities of EC, ECG, EGC, EGCG, theaflavins and theanine compounds were also demonstrated. The anticancer behavior of EGCG on MCF7 cells was shown in several studies (89–91, 97). Tran et al. (90) revealed that EGCG suppressed the expressions of heat shock proteins (HSP70 and HSP90), increasing cell resistance to apoptosis, in MCF7 cells by inhibiting their heat shock transcription factors (HSF1 and HSF2). In addition, Machado et al. (92) exhibited that caffeine reduced the cell viability of MCF7 cells by inducing apoptosis through oxidative stress. The anticancer activity of gallic acid on MCF7 cells was also verified in the studies of Aborehab et al. (93), Rezaei-Seresht et al. (94) and Wang et al. (95).

Taken together, these findings indicate that the anticancer activity of black tea waste fiber-derived instant teas was influenced by both extraction-dependent composition and cell-line-specific sensitivity. The higher catechin and condensed phenolic contents of HI were consistent with its stronger inhibitory effect on glioblastoma cells, particularly U87MG. However, the higher response of MCF7 cells to CI showed that cytotoxic activity was not simply proportional to the major phenolic fractions. This suggests that the biological activity of the instant tea samples may arise from the combined contribution of multiple compounds, including catechins, theaflavins, gallic acid and caffeine, as well as possible compound interactions and matrix-related effects. Therefore, the observed anticancer activity should be interpreted as a cell-line-dependent response associated with the overall phytochemical profile of the instant tea samples rather than with a single dominant compound.

## 4 Conclusion

This study demonstrated, for the first time, the anticancer potential of instant teas produced from black tea waste fibers. The instant tea extracts exhibited cytotoxic activity against prostate cancer cell lines (PC3, 22Rv1, and LNCaP) *in vitro*, with LNCaP identified as the most resistant cell line, followed by 22Rv1 and PC3. In terms of extraction efficiency, hot extraction resulted in significantly higher levels of catechins (EC, ECG, EGC and EGCG), condensed phenolics (theaflavins and thearubigins) and theanine, which was also associated with stronger cytotoxic effects on glioblastoma multiforme cells (U87MG and A172). Among these, A172 cells exhibited greater resistance compared to U87MG cells. In contrast, cold extraction showed comparable efficiency in the extraction of gallic acid and caffeine (*p* > 0.05) and demonstrated stronger inhibitory effects on MCF7 breast cancer cells. These findings suggest that bioactive compounds other than the major catechins and phenolic fractions may also contribute to the observed anticancer activity of cold-extracted instant teas. Overall, the results highlight that black tea waste fibers can serve as a valuable raw material for the production of instant tea with a considerable bioactive profile and potential biological activity. Further studies are needed to identify the compounds responsible for the enhanced anticancer activity observed in cold-extracted samples and to better understand the mechanisms underlying these effects.

## Data Availability

The raw data supporting the conclusions of this article will be made available by the authors, without undue reservation.

## References

[1] Food and Agricultural Organization. International tea market: market Situation, Prospects and Emerging Issues. (2022).

[2] Zhang L Ho CT Zhou J Santos JS Armstrong L Granato D. Chemistry and biological activities of processed Camellia sinensis teas: a comprehensive review. Compr Rev Food Sci Food Saf. (2019) 18:1474–1495. doi: 10.1111/1541-4337.12479 33336903

[3] Debnath B Haldar D Purkait MK. Potential and sustainable utilization of tea waste: a review on present status and future trends. J Environ Chem Eng. (2021) 9:106179. doi: 10.1016/j.jece.2021.106179

[4] Sayar NA Durmaz Şam S Pinar O Serper D Sarıyar Akbulut B Kazan D . Techno-economic analysis of caffeine and catechins production from black tea waste. Food Bioprod Process. (2019) 118:1–12. doi: 10.1016/j.fbp.2019.08.014

[5] Kraujalyte V Pelvan E Alasalvar C. Volatile compounds and sensory characteristics of various instant teas produced from black tea. Food Chem. (2016) 194:864–72. doi: 10.1016/J.FOODCHEM.2015.08.051, 26471629

[6] Chandini SK Jaganmohan Rao L Subramanian R. Influence of extraction conditions on polyphenols in black tea extracts. Int J Food Sci Technol. (2011) 46:879–86. doi: 10.1111/J.1365-2621.2011.02576.X

[7] Liu HY Liu Y Mai YH Guo H He XQ Xia Y . Phenolic content, main flavonoids, and antioxidant capacity of instant sweet tea (Lithocarpus litseifolius [Hance] Chun) prepared with different raw materials and drying methods. Foods. (2021) 10:1930. doi: 10.3390/FOODS10081930, 34441707 PMC8394704

[8] Carvalho Rodrigues V Silva MV Santos AR Zielinski AAF Haminiuk CWI. Evaluation of hot and cold extraction of bioactive compounds in teas. Int J Food Sci Technol. (2015) 50:2038–45. doi: 10.1111/IJFS.12858

[9] Hajiaghaalipour F Sanusi J Kanthimathi MS. Temperature and time affect antioxidant properties of tea infusions. J Food Sci. (2016) 81:H246–54. doi: 10.1111/1750-3841.13149, 26613545

[10] Venditti E Bacchetti T Tiano L Carloni P Greci L Damiani E. Hot vs. cold water steeping of different teas: do they affect antioxidant activity? Food Chem. (2010) 119:1597–604. doi: 10.1016/J.FOODCHEM.2009.09.049

[11] Yang DJ Hwang LS Lin JT. Effects of steeping methods on tea composition. J Chromatogr A. (2007) 1156:312–20. doi: 10.1016/J.CHROMA.2006.11.088, 17161409

[12] Khokhar S Magnusdottir SGM. Total phenol, catechin, and caffeine contents of teas commonly consumed in the United Kingdom. J Agric Food Chem. (2002) 50:565–70. doi: 10.1021/JF010153L, 11804530

[13] Kelebek H. LC-DAD–ESI-MS/MS characterization of phenolic constituents in Turkish black tea: effect of infusion time and temperature. Food Chem. (2016) 204:227–38. doi: 10.1016/J.FOODCHEM.2016.02.132, 26988497

[14] Polat A Kalcıoğlu Z Müezzinoğlu N. Effect of infusion time on black tea quality, mineral content and sensory properties prepared using traditional Turkish infusion method. Int J Gastron Food Sci. (2022) 29:100559. doi: 10.1016/J.IJGFS.2022.100559

[15] Mu W Zhang T Jiang B. An overview of biological production of L-theanine. Biotechnol Adv. (2015) 33:335–42. doi: 10.1016/J.BIOTECHADV.2015.04.004, 25871834

[16] Serpen A Pelvan E Alasalvar C Mogol BA Yavuz HT Gökmen V . Nutritional and functional characteristics of seven grades of black tea produced in Turkey. J Agric Food Chem. (2012) 60:7682–9. doi: 10.1021/JF302058D, 22800200

[17] Pedro AC Maciel GM Ribeiro VR Haminiuk CWI. Fundamental and applied aspects of catechins from different sources: a review. Int J Food Sci Technol. (2020) 55:429–42. doi: 10.1111/ijfs.14371

[18] Yang CS Wang H. Cancer preventive activities of tea catechins. Molecules. (2016) 21:1679. doi: 10.3390/MOLECULES21121679, 27941682 PMC6273642

[19] Wei Y Jin TY Wei K Zuo XB Yang XF Wang KB . Comparison of chemical profiles, dissolution patterns, sensory evaluation and bioactivity of selenium-enriched green tea and black tea: influence of brewing conditions. Food Med Homol. (2026) 3:9420110. doi: 10.26599/FMH.2026.9420110

[20] Zhao YL Tang R Liu S Han ST Feng J Chi KX . Effects of urolithin A-producing *Streptococcus thermophilus* FUA329 fermentation on the composition and antioxidant bioactivities of black tea. Food Med Homol. (2025) 2:9420041. doi: 10.26599/FMH.2025.9420041

[21] Bray F Ferlay J Soerjomataram I Siegel RL Torre LA Jemal A. Global cancer statistics 2018: GLOBOCAN estimates of incidence and mortality worldwide for 36 cancers in 185 countries. CA Cancer J Clin. (2018) 68:394–424. doi: 10.3322/CAAC.21492 30207593

[22] Briguglio G Costa C Pollicino M Giambò F Catania S Fenga C. Polyphenols in cancer prevention: new insights. Int J Funct Nutr. (2020) 1:9. doi: 10.3892/IJFN.2020.9

[23] Rawla P. Epidemiology of prostate cancer. World J Oncol. (2019) 10:63–89. doi: 10.4021/wjon.v10i2.1191, 31068988 PMC6497009

[24] Zheng H Yan T Han Y Wang Q Zhang G Zhang L . Prognostic assessment in glioblastoma. Clin Genet. (2022) 102:359–68. doi: 10.1111/CGE.14200, 35882630

[25] Wilkinson L Gathani T. Understanding breast cancer as a global health concern. Br J Radiol. (2022) 95:20211033. doi: 10.1259/BJR.20211033, 34905391 PMC8822551

[26] Alserihi RF Mohammed MRS Kaleem M Khan MI Sechi M Zughaibi TA . Comparative efficacy of epigallocatechin gallate and its nano-formulation in prostate cancer 3D spheroids model. J King Saud Univ Sci. (2023) 35:102627. doi: 10.1016/J.JKSUS.2023.102627

[27] Elhag R Mazzio EA Soliman KFA. The effect of silibinin in enhancing toxicity of temozolomide and etoposide in resistant glioma cell lines. Anticancer Res. (2015) 35:1263–9. 25750273 PMC4355951

[28] Andleeb A Asghar S Zaman G Tariq M Mehmood A Nadeem M . A systematic review of biosynthesized metallic nanoparticles as a promising anti-cancer strategy. Cancers. (2021) 13:2818. doi: 10.3390/cancers13112818, 34198769 PMC8201057

[29] Cadoná FC Dantas RF Mello GH Silva FP Jr. Natural products targeting cancer hallmarks: caffeine, theobromine, and catechin. Crit Rev Food Sci Nutr. (2022) 62:7222–41. doi: 10.1080/10408398.2021.1913091, 33890518

[30] Luo T Jiang JG. Anticancer effects of theaflavins. J Agric Food Chem. (2021) 69:15052–65. doi: 10.1021/ACS.JAFC.1C05313, 34878780

[31] Shojaei-Zarghani S Rafraf M Yari-Khosroushahi A. Theanine and cancer: a systematic review. Phytother Res. (2021) 35:4782–94. doi: 10.1002/PTR.7110, 33891786

[32] Yang G Meng Q Shi J Zhou M Zhu Y You Q . Functional tea products: health benefits and processing. Compr Rev Food Sci Food Saf. (2023) 22:1686–721. doi: 10.1111/1541-4337.13127, 36856036

[33] Cifte NE Taskin E Gorgisen G Namli S Yetgin MM Gurleyik A . Valorization of black tea waste fibers into instant teas and characterization of their bioactive profile and physicochemical properties. ACS Food Sci Technol. (2025) 5:162–74. doi: 10.1021/acsfoodscitech.4c00665

[34] Murugesh CS Rastogi NK Subramanian R. Athermal extraction of green tea: optimisation and kinetics of extraction of polyphenolic compounds. Innov Food Sci Emerg Technol. (2018) 50:207–16. doi: 10.1016/J.IFSET.2018.06.005

[35] Obanda M Owuor PO Mang’oka R Kavoi MM. Changes in thearubigin fractions and theaflavin levels due to variations in processing conditions and their influence on black tea liquor brightness and total colour. Food Chem. (2004) 85:163–73. doi: 10.1016/S0308-8146(02)00183-8

[36] Özdemir F Şahin Nadeem H Akdoğan A Dinçer C Topuz A. Effect of altitude, shooting period, and tea grade on the catechins, caffeine, theaflavin, and thearubigin of Turkish black tea. Turk J Agric For. (2018) 42:334–40. doi: 10.3906/tar-1710-21

[37] Alasalvar C Pelvan E Özdemir KS Kocadagìli T Mogol BA Pasli AA . Compositional, nutritional, and functional characteristics of instant teas produced from low- and high-quality black teas. J Agric Food Chem. (2013) 61:7529–36. doi: 10.1021/JF4015137, 23837397

[38] Someswararao C Srivastav PP. A novel technology for production of instant tea powder from the existing black tea manufacturing process. Innov Food Sci Emerg Technol. (2012) 16:143–7. doi: 10.1016/j.ifset.2012.05.005

[39] Fernández PL Martin MJ González AG Pablos F. HPLC determination of catechins and caffeine in tea. Differentiation of green, black and instant teas. Analyst. (2000) 125:421–5. doi: 10.1039/A909219F, 10829341

[40] Friedman M Mackey BE Kim HJ Lee IS Lee KR Lee SU . Structure–activity relationships of tea compounds against human cancer cells. J Agric Food Chem. (2006) 55:243–53. doi: 10.1021/JF062276H, 17227049

[41] Tong T Liu YJ Kang J Zhang CM Kang SG. Antioxidant activity and main chemical components of a novel fermented tea. Molecules. (2019) 24:2917. doi: 10.3390/MOLECULES24162917, 31408939 PMC6720624

[42] Cabrera C Giménez R López MC. Determination of tea components with antioxidant activity. J Agric Food Chem. (2003) 51:4427–35. doi: 10.1021/JF0300801, 12848521

[43] Boros K Jedlinszki N Csupor D. Theanine and caffeine content of infusions prepared from commercial tea samples. Pharmacogn Mag. (2016) 12:75–9. doi: 10.4103/0973-1296.176061, 27019564 PMC4787341

[44] Too J Wanyoko J Kinyanjui T Moseti K Wachira F. Quantitative estimation of γ-glutamylethylamide in commercially available made teas [*Camellia sinensis* (L.) O. Kuntze, Theaceae] in Kenya. Am J Plant Sci. (2016) 7:55–62. doi: 10.4236/AJPS.2016.71006

[45] Bieżanowska-Kopeć R Piatkowska E. Total polyphenols and antioxidant properties of selected fresh and dried herbs and spices. Appl Sci. (2022) 12:4876. doi: 10.3390/APP12104876

[46] Ramalho SA Nigam N Oliveira GB Oliveira PA Silva TOM Santos AGP . Effect of infusion time on phenolic compounds and caffeine content in black tea. Food Res Int. (2013) 51:155–61. doi: 10.1016/J.FOODRES.2012.11.031

[47] Vuong QV Golding JB Nguyen M Roach PD. Extraction and isolation of catechins from tea. J Sep Sci. (2010) 33:3415–28. doi: 10.1002/JSSC.201000438, 21049524

[48] Vuong QV Stathopoulos CE Golding JB Nguyen MH Roach PD. Optimum conditions for L-theanine extraction. J Sep Sci. (2011) 34:2468–74. doi: 10.1002/JSSC.201100401, 21735551

[49] Fuller M Rao NZ. Effect of time, roasting temperature, and grind size on caffeine and chlorogenic acid concentrations in cold brew coffee. Sci Rep. (2017) 7:1–9. doi: 10.1038/s41598-017-18247-4, 29269877 PMC5740146

[50] Damiani E Bacchetti T Padella L Tiano L Carloni P. Antioxidant activity of different white teas: comparison of hot and cold tea infusions. J Food Compos Anal. (2014) 33:59–66. doi: 10.1016/J.JFCA.2013.09.010

[51] Sun SL He GQ Yu HN Yang JG Borthakur D Zhang LC . Free Zn^2+^ enhances inhibitory effects of EGCG on the growth of PC‐3 cells. Mol Nutr Food Res. (2008) 52:465–71. doi: 10.1002/MNFR.200700172, 18324707

[52] Miyata Y Shida Y Hakariya T Sakai H. Anti-cancer effects of green tea polyphenols against prostate cancer. Molecules. (2019) 24:193. doi: 10.3390/MOLECULES24010193, 30621039 PMC6337309

[53] Yang J Yu H Sun S Zhang L Das UN Ruan H . Zn2+ interaction enhances EGCG effects. Biol Trace Elem Res. (2009) 131:298–310. doi: 10.1007/S12011-009-8362-5 19326061

[54] Fink SL Cookson BT. Apoptosis, pyroptosis, and necrosis: mechanistic description of dead and dying eukaryotic cells. Infect Immun. (2005) 73:1907–16. doi: 10.1128/IAI.73.4.1907-1916.2005, 15784530 PMC1087413

[55] Sun S Pan S Miao A Ling C Pang S Tang J . Black tea extracts induce apoptosis in prostate cancer cells. Oncol Rep. (2013) 30:763–72. doi: 10.3892/OR.2013.2504, 23715786

[56] Liu KC Ho HC Huang AC Ji BC Lin HY Chueh FS . Gallic acid provokes DNA damage and suppresses DNA repair gene expression in human prostate cancer PC‐3 cells. Environ Toxicol. (2013) 28:579–87. doi: 10.1002/TOX.20752, 21887735

[57] Villalpando-Rodriguez GE Gibson SB. Reactive oxygen species (ROS) regulates different types of cell death by acting as a rheostat. Oxidative Med Cell Longev. (2021) 2021:9912436. doi: 10.1155/2021/9912436, 34426760 PMC8380163

[58] Perez JE Nagle I Wilhelm C. Magnetic molding of tumor spheroids. Biofabrication. (2020) 13:015018. doi: 10.1088/1758-5090/abc670, 33126227

[59] Rahman Siddique H Nanda S Parray A Saleem M. Androgen receptor in human health: a potential therapeutic target. Curr Drug Targets. (2012) 13:1907–16. doi: 10.2174/138945012804545579, 23140299

[60] Kaur M Velmurugan B Rajamanickam S Agarwal R Agarwal C. Gallic acid exhibits anti-proliferative effects in prostate cancer. Pharm Res. (2009) 26:2133–40. doi: 10.1007/S11095-009-9926-Y, 19543955 PMC2741017

[61] Eom DW Lee JH Kim YJ Hwang GS Kim SN Kwak JH . Synergistic effect of curcumin on epigallocatechin gallate-induced anticancer action in PC3 prostate cancer cells. BMB Rep. (2015) 48:461–6. doi: 10.5483/BMBRep.2015.48.8.216, 25441423 PMC4576954

[62] Bakhshandeh N Mohammadi M Mohammadi P Nazari E Damchi M Khodabandelu S . Increased expression of androgen receptor and PSA genes in LNCaP (prostate cancer) cell line due to high concentrations of EGCG, an active ingredient in green tea. Horm Mol Biol Clin Investig. (2022) 44:181–6. doi: 10.1515/HMBCI-2022-0054, 36578191

[63] Nam S Smith DM Dou QP. Tea polyphenols inhibit proteasome activity. J Biol Chem. (2001) 276:13322–30. doi: 10.1074/JBC.M004209200, 11278274

[64] Oeckinghaus A Ghosh S. The NF-κB family of transcription factors and its regulation. Cold Spring Harb Perspect Biol. (2009) 1:a000034. doi: 10.1101/cshperspect.a000034, 20066092 PMC2773619

[65] Chen BH Hsieh CH Tsai SY Wang CY Wang CC. Anticancer effects of epigallocatechin-3-gallate nanoemulsion on lung cancer cells through the activation of AMP-activated protein kinase signaling pathway. Sci Rep. (2020) 10:1–11. doi: 10.1038/s41598-020-62136-2, 32198390 PMC7083948

[66] Lin JK Lee HH Ho CT. Theaflavins inhibit prostate cancer cell growth. ACS Symp Ser. (2008) 987:160–70. doi: 10.1021/BK-2008-0987.CH010

[67] Russell LH Mazzio E Badisa RB Zhu ZP Agharahimi M Oriaku ET . Autoxidation of gallic acid induces ROS-dependent death. Anticancer Res. (2012) 32, 1595–1602. 22593437 PMC3356927

[68] Wong SC Kamarudin MNA Naidu R. Anticancer mechanism of flavonoids on high-grade adult-type diffuse gliomas. Nutrients. (2023) 15:797. doi: 10.3390/NU15040797, 36839156 PMC9964830

[69] Hopkins BD Hodakoski C Barrows D Mense SM Parsons RE. PTEN function: the long and the short of it. Trends Biochem Sci. (2014) 39:183–90. doi: 10.1016/J.TIBS.2014.02.006, 24656806 PMC4043120

[70] Knowles MA Platt FM Ross RL Hurst CD. PI3K pathway in cancer. Cancer Metastasis Rev. (2009) 28:305–16. doi: 10.1007/S10555-009-9198-3, 20013032 PMC2797439

[71] Song MS Salmena L Pandolfi PP. PTEN tumour suppressor regulation. Nat Rev Mol Cell Biol. (2012) 13:283–96. doi: 10.1038/nrm3330, 22473468

[72] Mantovani F Collavin L Del Sal G. Mutant p53 as a guardian of the cancer cell. Cell Death Differ. (2019) 26:199–212. doi: 10.1038/S41418-018-0246-9, 30538286 PMC6329812

[73] Marei HE Althani A Afifi N Hasan A Caceci T Pozzoli G . p53 signaling in cancer progression and therapy. Cancer Cell Int. (2021) 21:703. doi: 10.1186/S12935-021-02396-8, 34952583 PMC8709944

[74] Mirzayans R Andrais B Scott A Murray D. New insights into p53 signaling and cancer cell response to DNA damage. J Biomed Biotechnol. (2012) 2012:1–16. doi: 10.1155/2012/170325, 22911014 PMC3403320

[75] Das A Banik NL Ray SK. Flavonoids activated caspases for apoptosis in human glioblastoma T98G and U87MG cells but not in human normal astrocytes. Cancer. (2010) 116:164–76. doi: 10.1002/CNCR.24699, 19894226 PMC3159962

[76] Grube S Ewald C Kögler C McLean AL Kalff R Walter J. CNS concentrations of EGCG induce stress in glioblastoma cells. Nutr Cancer. (2018) 70:1145–58. doi: 10.1080/01635581.2018.1495239, 30198785

[77] Shervington A Pawar V Menon S Thakkar D Patel R. Sensitization of glioma cells to chemotherapy by catechin. Mol Biol Rep. (2009) 36:1181–6. doi: 10.1007/S11033-008-9295-3, 18581255

[78] Yang JT Lee IN Chen CH Lu FJ Chung CY Lee MH . Gallic acid enhances the anti-cancer effect of temozolomide in human glioma cell line via inhibition of Akt and p38-MAPK pathway. Processes. (2022) 10:448. doi: 10.3390/PR10030448

[79] Li N Zhang P Kiang KMY Cheng YS Leung GKK. Caffeine sensitizes U87-MG human glioblastoma cells to temozolomide through mitotic catastrophe by impeding G2 arrest. Biomed Res Int. (2018) 2018:1–10. doi: 10.1155/2018/5364973, 30050935 PMC6046144

[80] Siegelin MD Habel A Gaiser T. Epigalocatechin-3-gallate (EGCG) downregulates PEA15 and thereby augments TRAIL-mediated apoptosis in malignant glioma. Neurosci Lett. (2008) 448:161–5. doi: 10.1016/J.NEULET.2008.10.036, 18948169

[81] Szliszka E Mazur B Zdowicz G Czuba ZP Król W. TRAIL-induced apoptosis in cancer cells. Folia Histochem Cytobiol. (2009) 47:579–85. doi: 10.2478/V10042-009-0111-2, 20430723

[82] Sachinidis A Seul C Seewald S Ahn HY Ko Y Vetter H. Green tea compounds inhibit glioblastoma signaling. FEBS Lett. (2000) 471:51–5. doi: 10.1016/S0014-5793(00)01360-0, 10760511

[83] Hou Z Lambert JD Chin KV Yang CS. Effects of tea polyphenols on signal transduction pathways related to cancer chemoprevention. Mutat Res. (2004) 555:3–19. doi: 10.1016/J.MRFMMM.2004.06.040, 15476848

[84] Way TD Lin HY Hua KT Lee JC Li WH Lee MR . Beneficial effects of different tea flowers against human breast cancer MCF-7 cells. Food Chem. (2009) 114:1231–6. doi: 10.1016/J.FOODCHEM.2008.10.084

[85] González SCE Bolaina-Lorenzo E Pérez-Trujillo JJ Puente-Urbina BA Rodríguez-Fernández O Fonseca-García A . Antibacterial and anticancer activity of ZnO with different morphologies: a comparative study. 3 Biotech. (2021) 11:68. doi: 10.1007/s13205-020-02611-9, 33489685 PMC7806688

[86] Jaragh-Alhadad L Behbehani H Karnik S. Cancer targeted drug delivery using active low-density lipoprotein nanoparticles encapsulated pyrimidines heterocyclic anticancer agents as microtubule inhibitors. Drug Deliv. (2022) 29:2759–72. doi: 10.1080/10717544.2022.2117435, 36029014 PMC9427048

[87] Wang X Li X Ito A Watanabe Y Tsuji NM. Rod-shaped and fluorine-substituted hydroxyapatite free of molecular immunopotentiators stimulates anti-cancer immunity in vivo. Chem Commun. (2016) 52:7078–81. doi: 10.1039/C6CC02848A, 27121009

[88] Koňariková K Ježovičová M Keresteš J Gbelcová H Ďuračková Z Žitňanová I. Anticancer effect of black tea extract. Springerplus. (2015) 4:127. doi: 10.1186/S40064-015-0871-4, 25825685 PMC4374083

[89] Schröder L Marahrens P Koch JG Heidegger H Vilsmeier T Phan-Brehm T . Effects of green tea, matcha tea and their components epigallocatechin gallate and quercetin on MCF‑7 and MDA-MB-231 breast carcinoma cells. Oncol Rep. (2019) 41:387–96. doi: 10.3892/OR.2018.6789 30320348

[90] Tran PLC Kim SA Choi HS Yoon JH Ahn SG. Epigallocatechin-3-gallate suppresses the expression of HSP70 and HSP90 and exhibits anti-tumor activity in vitro and in vivo. BMC Cancer. (2010) 10:276. doi: 10.1186/1471-2407-10-276, 20537126 PMC2927993

[91] Wang P Henning SM Heber D. Limitations of MTT and MTS-based assays for measurement of antiproliferative activity of green tea polyphenols. PLoS One. (2010) 5:e10202. doi: 10.1371/JOURNAL.PONE.0010202, 20419137 PMC2855713

[92] Machado KL Marinello PC Silva TNX Silva CFN Luiz RC Cecchini R . Oxidative stress in caffeine action on the proliferation and death of human breast cancer cells MCF-7 and MDA-MB-231. Nutr Cancer. (2021) 73:1378–88. doi: 10.1080/01635581.2020.1795693, 32691663

[93] Aborehab NM Elnagar MR Waly NE. Gallic acid potentiates the apoptotic effect of paclitaxel and carboplatin via overexpression of Bax and P53 on the MCF-7 human breast cancer cell line. J Biochem Mol Toxicol. (2021) 35:e22638. doi: 10.1002/JBT.22638, 33002289

[94] Rezaei-Seresht H Cheshomi H Falanji F Movahedi-Motlagh F Hashemian M Mireskandari E. Cytotoxic activity of gallic acid against MCF-7 cells. Avicenna J Phytomed. (2019) 9:574–86. doi: 10.22038/AJP.2019.13475, 31763216 PMC6823530

[95] Wang K Zhu X Zhang K Zhu L Zhou F. Gallic acid induced anticancer effect in MCF-7 cells. J Biochem Mol Toxicol. (2014) 28:387–93. doi: 10.1002/JBT.21575, 24864015

[96] Yang J Liu RH. The phenolic profiles and antioxidant activity in different types of tea. Int J Food Sci Technol. (2013) 48:163–71. doi: 10.1111/J.1365-2621.2012.03173.X

[97] Zhang SY Ma XF Zheng CG Wang Y Cao XL Tian WX. Catechin-derived inhibitors on MCF-7 cells. J Enzyme Inhib Med Chem. (2009) 24:623–31. doi: 10.1080/14756360802319678, 18671164

