# Supplementary figures and images for: Functional instant tea from black tea waste fiber: effects of extraction conditions on bioactive composition and in vitro anticancer activity
Source: Front Nutr. 2026 Jun 30;13:1861937. doi: 10.3389/fnut.2026.1861937 (PMC13364970; doi:10.3389/fnut.2026.1861937)

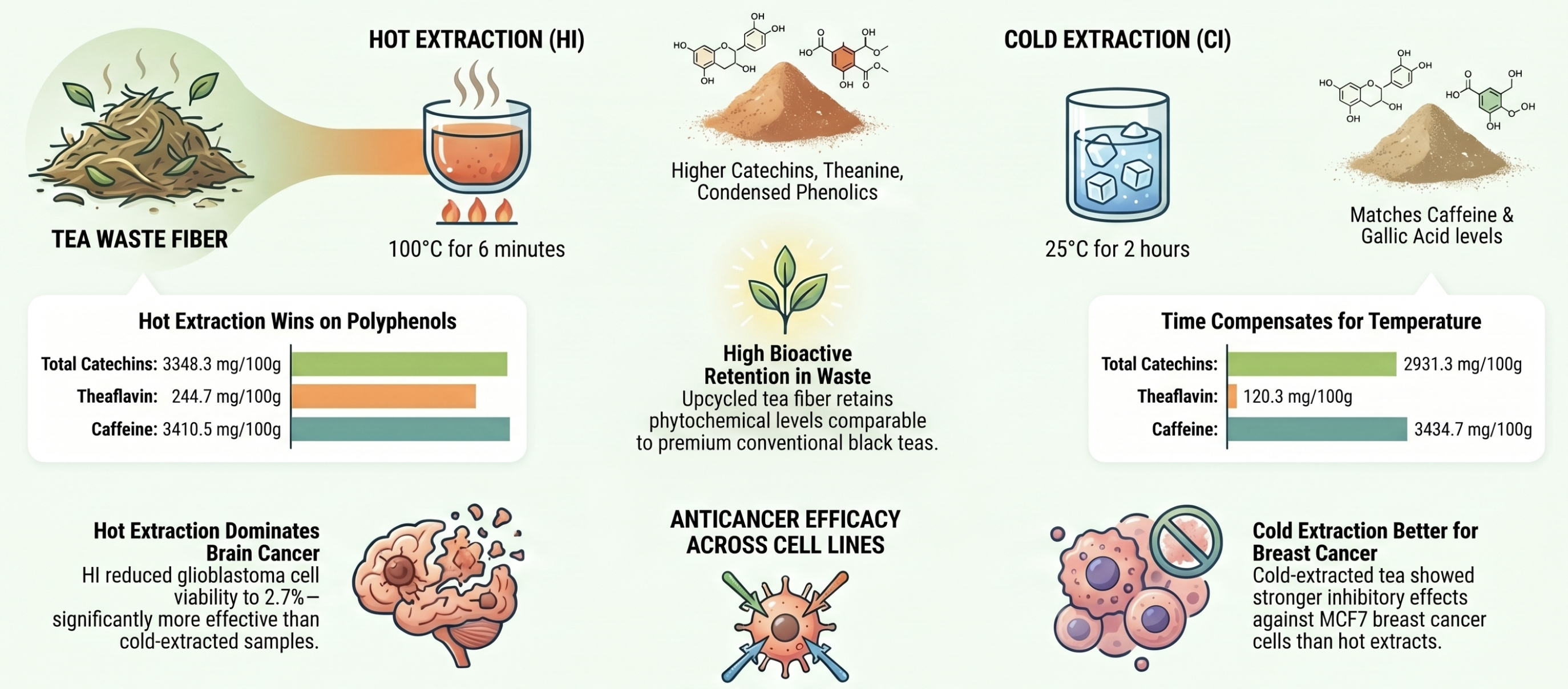

Supplement: Supplementary file 1 [file Image_1.PNG]
